# *In vitro* susceptibility to fosfomycin in clinical and environmental extended-spectrum beta-lactamase producing and/or ciprofloxacin-non-susceptible *Escherichia coli* isolates

**DOI:** 10.1590/S1678-9946202466005

**Published:** 2024-02-05

**Authors:** Victoria Stadler Tasca Ribeiro, Larissa Bail, Carmen Antonia Sanches Ito, Ana Paula de Andrade, Lavinia Nery Villa Stangler Arend, Paula Hansen Suss, Keite da Silva Nogueira, Haniel Siqueira Mortagua Walflor, Helisson Faoro, Lia Carolina Soares de Medeiros Kuczera, Fernando José Vicenzi, Felipe Francisco Tuon

**Affiliations:** 1 Pontifícia Universidade Católica do Paraná, Escola de Medicina, Programa de Pós-Graduação em Ciências da Saúde, Laboratório de Doenças Infecciosas Emergentes, Curitiba, Paraná, Brazil; 2 Universidade Estadual de Ponta Grossa do Paraná, Divisão de Microbiologia, Ponta Grossa, Paraná, Brazil; 3 Laboratório Central do Estado do Paraná, São José dos Pinhais, Paraná, Brazil; 4 Universidade Federal do Paraná, Departamento de Patologia Básica, Curitiba, Paraná, Brazil; 5 Fiocruz Paraná, Instituto Carlos Chagas, Laboratório de Ciências e Tecnologias Aplicadas à Saúde, Curitiba, Paraná, Brazil; 6 Fiocruz Paraná, Instituto Carlos Chagas, Laboratório de Biologia Celular, Curitiba, Paraná, Brazil; 7 Laboratório Municipal de Curitiba, Curitiba, Paraná, Brazil

**Keywords:** Gram-negative bacilli, Escherichia coli, Fosfomycin, ESBL, Environment

## Abstract

Extended-spectrum beta-lactamase producing and ciprofloxacin-non-susceptible *Escherichia coli* are clinical and environmental issues. We evaluated the susceptibility profile of fosfomycin in non-susceptible *E. coli* isolated from urine and the environment. We measured the activity of fosfomycin against 319 and 36 *E. coli* strains from urine and environmental isolates, respectively, collected from rivers. Fosfomycin resistance profiles were investigated using the minimal inhibitory concentration (MIC), according to the Clinical and Laboratory Standards Institute (CLSI) and the European Committee for Antimicrobial Susceptibility Testing (EUCAST) guidelines. Antibiotic susceptibility testing revealed that 5% and 6.6% of urine samples were non-susceptible to fosfomycin according to CLSI and EUCAST guidelines, respectively. The fosfomycin MIC50/90 was 0.5/4 mg/L. Of the 36 *E. coli* isolates from river water, 11.1% and 13,8% were non-susceptible to fosfomycin according to CLSI and EUCAST, respectively (range ≤0.25 ≥512 mg/L). All the isolates with MIC ≥512 mg/L for fosfomycin showed the *fos*A3 gene. Fosfomycin resistance was more frequent in the environment than in clinical samples.

## INTRODUCTION

Urinary tract infections (UTIs) are a prevalent occurrence among both outpatients and individuals suffering from bacterial infections, with a pronounced impact on women^1^. Uropathogenic *Escherichia coli* is the main causative agent of uncomplicated (acute cystitis) and complicated (pyelonephritis) conditions^2,3^. Uncomplicated UTIs account for most antibiotic prescriptions in primary care settings worldwide, and treatment continues to be predominantly empirical^4,5^. Fluoroquinolones (e.g., ciprofloxacin and norfloxacin) and oral β-lactams (e.g., amoxicillin-clavulanate and cephalexin) have been widely used to treat these infections, contributing to the selection of drug-resistant strains^6,7^. Considering the increasing resistance to fluoroquinolones and β-lactams that is currently being observed^8^, the Infectious Diseases Society of America (IDSA) recommends these drugs as second-line therapies^9^.

Over the past decade, the primary challenge in treating UTIs has been the resistance observed in third- and fourth-generation cephalosporins, often associated with extended-spectrum beta-lactamases (ESBL)^10,11^. This mechanism of resistance is mediated by plasmids that confer resistance to other antibiotics^12^. Bacteria harboring resistance genes, particularly ESBLs, have been detected in river water, and their presence during activities such as irrigation and recreational use can potentially lead to colonization or infection in both humans and animals^13^.

Nitrofurantoin, trimethoprim-sulfamethoxazole, fosfomycin, and pivmecillinam are currently recommended as first-line therapies for treating acute uncomplicated cystitis in women. These options are considered suitable if local resistance rates are below 20%, or if the specific infecting strain is confirmed to be susceptible to these medications^9^. Hence, obtaining local susceptibility patterns is a crucial step in selecting these antibiotics for accurate empirical therapy.

Fosfomycin, a bactericidal antimicrobial drug discovered in 1969, has emerged as one of the most active drugs for uncomplicated UTIs^14^. Fosfomycin can be found in high concentrations in the urinary tract, and due to its single-dose oral administration and minimal side effects, it has been increasingly prescribed. This study aimed to assess the susceptibility profile of *E. coli* isolates to fosfomycin, including those carrying ESBL and non-susceptible to ciprofloxacin, from both urinary samples and the local environment in a city in southern Brazil.

## MATERIALS AND METHODS

This study evaluated the susceptibility of fosfomycin in 319 urinary *E. coli* strains isolated from May to September 2020 from 10 sanitary districts of Curitiba city, Parana State, Brazil, and 36 environmental isolates of *E. coli* collected from the main rivers of the Curitiba’s watershed (Atuba, Belem, Ribeirao dos Padilha, Barigui, Passauna, and Iguacu) from February to August 2021. Curitiba is a city located in Parana State, Brazil, with an estimated population of 1,963,726 inhabitants in an area of 434,892 km^2^ and 96.3% of adequate sanitation. The city is divided into 10 sanitary districts, defined as a geographical area that contains the population’s epidemiological and social characteristics and their necessities. Additionally, Curitiba presents a subtropical climate (according to the Köppen-Geiger’s classification scheme), with an annual precipitation of approximately 1,600 mm^15^.

### Clinical and environmental isolates

From May 2020 to September 2020, 1,049 *E. coli* strains were identified in the microbiology section of the Municipal Laboratory of Curitiba. In total, 319 non-duplicate *E. coli* from clinical outpatients urine samples were selected based on ciprofloxacin susceptibility and ESBL detection; 303 were non-susceptible to ciprofloxacin (88 ESBL-positive and 215 ESBL-negative), and 16 were ciprofloxacin susceptible (ESBL-positive). *E. coli* was identified using chromID^™^ CPS^®^ Elite (BioMérieux, Marcy l’Etoile, France). Ciprofloxacin susceptibility testing and ESBL detection were performed using a VITEK^®^ 2 AST-N238 card (BioMérieux, Marcy l’Etoile, France).

**Figure 1:**
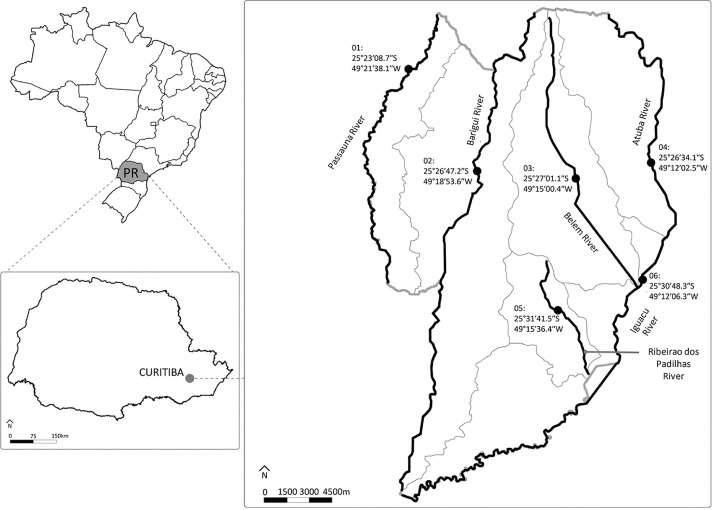
Points of water collection in the main rivers of the six watersheds of Curitiba: 01) Passauna River, 02) Barigui River, 03) Belem River, 04) Atuba River, 05) Ribeirao dos Padilhas River, and 06) Iguacu River.

A total of 36 non-duplicate *E. coli* isolates from the six main rivers of each watershed of Curitiba (environmental isolates) were selected based on ciprofloxacin susceptibility and ESBL detection (Figure 1 shows the sites of collection, considered as hotspots). First, 100 mL of river water from each site was collected in sterile flasks and placed on ice until processing (on the same day, no more than 2 h after collection). Subsequently, 100 mL of water was filtered through a 0.45 µm filter membrane (Merck Millipore, Darmstadt, Germany) and then added to EC Broth (Merck, San Luis, MI) at 36 °C for 24 h. After that, 100 µL of the resulting broth with observable growth was transferred to 9.9 mL of Mueller Hinton Cation Adjusted broth, supplemented with 1 mg/L of ciprofloxacin (Isofarma, Goiania, Brazil), aiming to select ciprofloxacin-non-susceptible *E. coli*. Simultaneously, another 100 µL of the initial broth was transferred to 9.9 mL of Mueller Hinton Cation Adjusted broth supplemented with 4 mg/L of ceftriaxone (ABL, Sao Paulo, Brazil) to select *E. coli* ESBL. Broth supplemented with ciprofloxacin or ceftriaxone were incubated at 36 °C for 24 h. After incubation, 10 µL was streaked on Chromocult^®^ Coliform Agar (Merck Millipore, Darmstadt, Germany) and incubated at 36 °C for 24 h. Purple colonies were selected and subjected to matrix-assisted laser desorption ionization time-of-flight mass spectrometry (MALDI-TOF-MS) (bioMerieux, Marcy l’Etoile, France) to confirm the identification of *E. coli*.

After this step, the colonies confirmed as *E. coli* in the previous step were subjected to testing on the Vitek^®^2 AST-N238 card (bioMerieux, Marcy l’Etoile, France) (Gram-negative susceptibility). Concomitantly, assessment of the ghost zone (conducted by the approximation of amoxicillin + clavulanic acid, ceftriaxone, cefepime, and ceftazidime disks) was conducted to confirm ESBL presence and/or ciprofloxacin resistance (evaluated in parallel by Vitek^®^ 2 AST-N238 card).

### Minimal inhibitory concentration (MIC)

Agar dilution (AD) quality control (QC) was performed using ATCC^®^ strains *E. coli* 25922 (Laborclin, Pinhais, Brazil), *Pseudomonas aeruginosa* 27853 (Laborclin, Pinhais, Brazil), and *Enterococcus faecalis* 29212 (Laborclin, Pinhais, Brazil). QC testing was performed in triplicate, and the results for each organism were within the acceptable limits of 99%. The VITEK^®^ 2 AST-N238 QC was performed weekly using ATCC^®^ strains, *Klebsiella pneumoniae* 700603 for the ESBL test, *E. coli* 25922, and *P. aeruginosa* 27853.

The clinical, environmental, and ATCC^®^ strains were initially subcultured on tryptic soy agar (18-24 h, 35 ± 2 °C). Fosfomycin MIC was determined using the AD method according to the Clinical and Laboratory Standards Institute (CLSI) M07-A10 guideline^16^ in the Laboratory of Emerging Infectious Diseases (LEID), School of Medicine, Pontificia Universidade Catolica do Parana, Curitiba city, Parana State, Brazil. Fosfomycin and D-glucose 6-phosphate were purchased from Sigma-Aldrich (St. Louis, MO, USA). AD tests for non-fastidious bacteria were performed using Mueller–Hinton agar (MHA) (BD Difco, Sparks, MD, USA). Glucose-6-phosphate was added to MHA at a 25 mg/L final concentration.

Briefly, 1 mL of each dilution and 1 mL of glucose-6-phosphate stock solution were added to 48 mL of agar in 45 to 50 °C in a Falcon tube. The tubes were mixed thoroughly and poured into 150 mm × 15 mm Petri plates. The agar solidified at room temperature, and plates were used immediately or stored in sealed plastic bags at 2 °C to 8 °C for up to five days. Two drug-free plates prepared from the base medium, with 1 mL of water and 1 mL of glucose-6-phosphate were used as growth controls, one at the beginning and the other at the end of inoculation. The bacterial suspension (0.5 McFarland standard) was prepared in saline by the direct colony suspension method using the Sensititre^™^ nephelometer (Thermo Fisher Scientific, MA), and 100 µL of each bacterium was dispensed in an ELISA plate without diluting the initial suspension. Using a Steers replicator with 1 mm pins, 0.1 µL to 0.2 µL were inoculated and plates incubated at 35 ± 2 °C for 16–20 h. The isolates and ATCC strains were evaluated in triplicate.

### Polymerase Chain Reaction for detecting fosA3 gene

Polymerase Chain Reaction (PCR) was employed to detect and evaluate the *fos*A3 gene—the most reported plasmid-mediated fosfomycin resistance gene among Enterobacterales, linked to the resistance dissemination worldwide. Firstly, an isolated colony was added to a nuclease free 0.2 mL microtube, and to the solution was later added 10 µL of OneTaq 2X (New England Biolabs, Ipswich, MA, USA). Then, 8 µL of nuclease-free water was poured, with 1 µL of forward and 1 µL of reverse primers (sequences: fosA3_FWD-II: GCATGCTGCAGGGATTGAATC and fosA3_REV-II: GCCAATCAAAAAAGACCATCCCC). The following parameters were used in the thermocycler: initial denaturation at 94 °C for 30 s, denaturation at 94 °C for 30 s, annealing at 60 °C for 30 s, extension at 68 °C for 45 s, and final extension at 68 °C for 5 min. Then, 5 to 10 µL of the PCR product (sample) was poured at an agarose gel at 2% with TBE buffer 1X. The run occurred for 40 min at 100 V. For control, an 1Kb Plus marker was used.

MICs for ciprofloxacin and ESBL detection were determined by experienced technicians using the VITEK^®^ 2 system (BioMérieux, Marcy l’Etoile, France) according to the manufacturer’s instructions. Ciprofloxacin MICs were interpreted following the European Committee for Antimicrobial Susceptibility Testing (EUCAST) guidelines as follows: susceptible (S) ≤ 0.25 mg/L; area of technical uncertainty (ATU) = 0.5; and resistant (R) ≥ 1 mg/L5. The VITEK^®^ 2 ESBL test is a confirmation test for clavulanic acid inhibited ESBL and uses cefepime (1 µg/mL), cefotaxime (0.5 µg/mL), and ceftazidime (0.5 µg/mL) with and without clavulanic acid (10 µg/mL, 4 µg/mL, and 4 µg/mL, respectively) to determine a positive or negative result. Fosfomycin MICs were interpreted following CLSI and EUCAST guidelines^17,18^. The fosfomycin CLSI breakpoints were S ≤64 mg/L, intermediate (I) = 128 mg/L, and R ≥ 256 mg/L. The EUCAST breakpoints were S ≤8 mg/L and R >8 mg/L. The PCR for *fos*A3 gene was evaluated qualitatively.

### Antibiotic prescribing evaluation

Data regarding the distribution of fosfomycin within the 10 sanitary districts were collected from the Municipal Health Department of Curitiba, which is part of the Brazilian Unified Health System (SUS). The data spanned from 2015, when fosfomycin was first introduced into the municipal health system, up to 2019. To assess the connection between fosfomycin-resistant isolates from clinical and environmental sources within the same geographic area, a correlation analysis was conducted. Statistical significance was considered if p-value < 0.05.

## RESULTS

**Figure 2:**
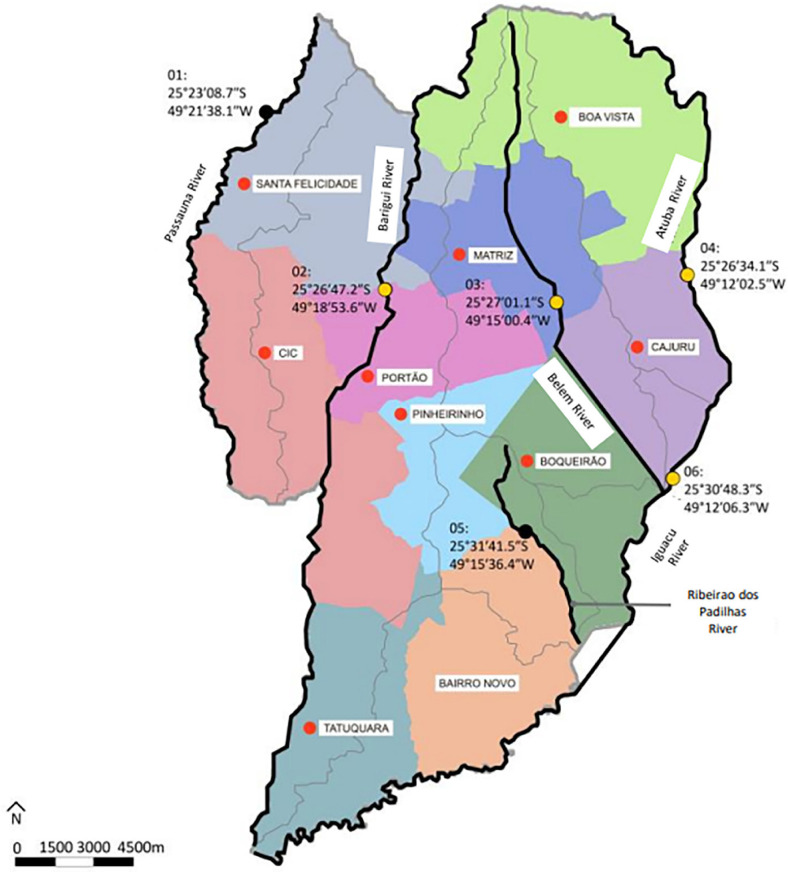
Detection and distribution of fosfomycin-resistant *Escherichia coli* according to sanitary districts (red dots) and rivers (yellow dots): 01) Passauna River, 02) Barigui River, 03) Belem River, 04) Atuba River, 05) Ribeirao dos Padilhas River, and 06) Iguacu River.

Of the 319 *E. coli* isolates from clinical urine samples from 10 sanitary districts, 16 (5%) and 21 (6.6%) were non-susceptible to fosfomycin according to CLSI and EUCAST guidelines, respectively. The fosfomycin MIC50/90 was 0.5/4 mg/L (range ≤ 0.25 to ≥ 512 mg/L). Only one sanitary district was absent of fosfomycin-non-susceptible *E. coli* (Bairro Novo) (Figure 2). Among the 303 non-susceptible to ciprofloxacin samples by EUCAST (MIC ≥ 0.5 mg/L), 17 (5.6%) had MIC ≥ 16 mg/L to fosfomycin, and among the 16 ciprofloxacin-sensitive samples, four (25%) had an MIC ≥ 16 mg/L to fosfomycin. Among the 104 ESBL-positive samples, 18 (17.3%) had an MIC ≥ 16 mg/L for fosfomycin, and among the 215 ESBL-negative samples, only three (1.4%) had an MIC ≥16 mg/L for fosfomycin (Table 1).

**Table 1:** Samples that showed resistance for fosfomycin among all the clinical urine samples. MIC results for ciprofloxacin (according to EUCAST), presence of ESBL and MIC results for fosfomycin (according to CLSI and EUCAST).

SAMPLE	SD	ESBL	Ciprofloxacin MIC (mg/L)		Fosfomycin MIC (mg/L)		
				EUCAST		CLSI	EUCAST
LMC1	Pinheirinho	Positive	1	R	>512	R	R
LMC4	Pinheirinho	Positive	≥4	R	>512	R	R
LMC11	Sta. Felicidade	Positive	≤0.25	S	>512	R	R
LMC17	Pinheirinho	Positive	≥4	R	>512	R	R
LMC22	Boa Vista	Positive	≤0.25	S	>512	R	R
LMC26	Portao	Positive	1	R	>512	R	R
LMC33	Pinheirinho	Positive	≥4	R	>512	R	R
LMC34	CIC	Positive	≥4	R	>512	R	R
LMC53	Tatuquara	Positive	≤0.25	S	128	I	R
LMC96	Boqueirao	Positive	≥4	R	16	S	R
LMC99	CIC	Negative	≥4	R	64	S	R
LMC134	CIC	Positive	≤0.25	S	>512	R	R
LMC151	Tatuquara	Positive	≥4	R	16	S	R
LMC170	Matriz	Positive	1	R	>512	R	R
LMC176	CIC	Positive	≥4	R	>512	R	R
LMC177	Boa Vista	Positive	2	R	>512	R	R
LMC210	Cajuru	Positive	≥4	R	>512	R	R
LMC232	Pinheirinho	Positive	≥4	R	64	S	R
LMC241	Cajuru	Positive	≥4	R	>512	R	R
LMC281	CIC	Negative	≥4	R	16	S	R
LMC286	Boqueirao	Negative	0.5	AUT	128	I	R

SD = sanitary district; MIC = minimum inhibitory concentration; ESBL = extended spectrum beta-lactamase; S = susceptible; R = resistant; I = intermediate; AUT = area of technical uncertainty; CIC = Curitiba industrial city.

Of the 36 *E. coli* isolates from the six rivers (environmental samples), *E. coli* non-susceptible to fosfomycin was not detected in only two rivers (Passauna and Ribeirao dos Padilhas). In total, 22 samples were screened as ESBL-positive, 28 as non-susceptible to ciprofloxacin, and 14 as ESBL-positive with ciprofloxacin resistance. Moreover, four and five samples (11.1%) were non-susceptible to fosfomycin by CLSI and EUCAST (range ≤ 0.25 to ≥ 512 mg/L), respectively. Among the five fosfomycin non-sensitive by EUCAST, all samples were ESBL-producers, and three were non-susceptible to ciprofloxacin (≥ 0.5 mg/L) (Table 2).

**Table 2:** Samples that showed resistance for fosfomycin among all the environmental samples. MIC results for ciprofloxacin (according to EUCAST), presence of ESBL and MIC results for fosfomycin (according to CLSI and EUCAST).

SAMPLE	RIVER	ESBL	Ciprofloxacin MIC (mg/L)		Fosfomycin MIC (mg/L)		
				EUCAST		CLSI	EUCAST
3-O	Belem	Positive	≥4	R	>512	R	R
31	Atuba	Positive	≥4	R	32	S	R
88	Iguacu	Positive	1	R	>512	R	R
114	Barigui	Positive	≤0.25	S	512	R	R
121	Belem	Positive	≤0.25	S	512	R	R

MIC = minimum inhibitory concentration; ESBL = extended-spectrum beta-lactamase; S = susceptible; R = resistant.

**Figure 3:**
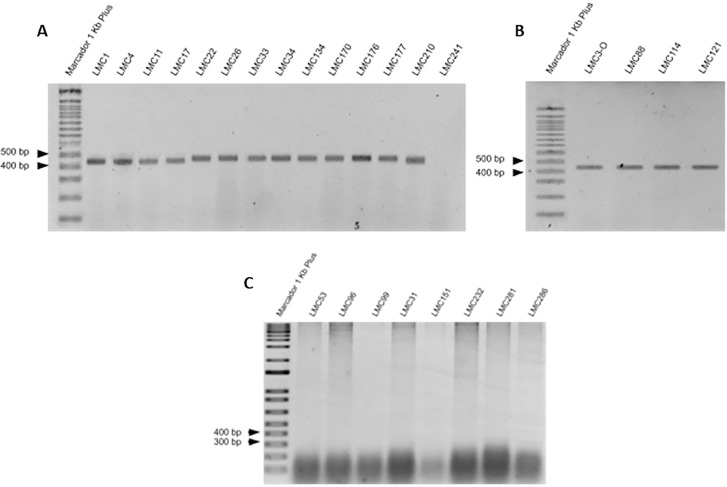
Isolates with MIC ≥ 512 mg/L for fosfomycin showing detection (A and B). Isolates with MIC values < 512 mg/L showed no gene amplification (C).

When analyzing the *fos*A3 gene by PCR, all the isolates with MIC ≥ 512 mg/L for fosfomycin showed detection (Figure 3A and 3B). Isolates with MIC values < 512 mg/L showed no gene amplification.

## DISCUSSION

*Escherichia coli* serves as a significant reservoir of resistance genes that have the potential to lead to treatment failures in both human and veterinary medicine^19^. In recent decades, increasing resistance genes in *E. coli* isolates have been observed, with a substantial portion of these resistance genes being acquired by horizontal gene transfer^20^. In the enterobacterial gene pool, *E. coli* acts as a donor and recipient of resistance genes and, thus, can acquire resistance genes from other bacteria, as well as pass on its resistance genes to other bacteria^21^.

Our study evaluated 319 clinical urine samples of 1,049 *E. coli* isolates from May 2020 to September 2020 at the Municipal Laboratory of Curitiba. Resistance to ciprofloxacin was higher than that of the ESBL-positive isolates (29% compared to 10%, respectively). The increasing emergence of fluoroquinolone-resistant *E. coli* has been reported worldwide, probably due to the excessive use of these antibiotics^22^. Shively *et al*.^23^ observed that, in 84% of cases, the prescription of ciprofloxacin for UTIs was inappropriate. In the same city as this study, a previous study from 2012 reported more than 20% fluoroquinolone resistance among *E. coli* ^11^. In addition, the prevalence of ESBL in *E. coli*, including TEM, SHV, and CTX-M types, from nosocomial and community-acquired UTIs is also increasing^7^.

Resistance to fosfomycin was observed in 16 (5%) and 21 (6.6%) clinical urine samples according to the CLSI and EUCAST breakpoints, respectively. These isolates were geographically scattered in nine of 10 sanitary districts, and the resistance profile was higher than that previously reported in the same city^24^. Interestingly, fosfomycin resistance was higher in the ESBL-producing isolates in our study than in a previous study by Tuon *et al*.^25^ in carbapenemase-producing *Klebsiella pneumoniae* 10 years ago. In France, given the increasing fluoroquinolone resistance, fosfomycin prescription increased by 41%, whereas norfloxacin and ciprofloxacin prescription reduced by 80% and 26%, respectively^26^. In Curitiba, the data on fosfomycin consumption is not readily available. However, the observed increase in resistance, as indicated by previous publications, could be one plausible hypothesis for this trend. All the isolates with MIC ≥512 mg/L for fosfomycin showed the *fos*A3 gene. This gene was firstly described in *Kluyvera georgiana* ^27^. This is the most common gene of fosfomycin resistance in the world, including in poultry^28^. The data we found are important to report the epidemiology of fosfomycin in Brazil.

The higher level of resistance to fosfomycin in isolates from the environment in comparison to clinical isolates (13.8% vs. 6.5%, respectively) was especially notable. Moreover, these environmental isolates were found in four out of the six main rivers in Curitiba city. The identification of mobile fosfomycin-resistance genes in isolates from various sources, including humans, animals, food, and the environment, has raised significant concerns regarding the potential for the dissemination of such bacteria, particularly *E. coli* and *Salmonella*, at the human-animal-environment interface^29^. To the best of our knowledge, no studies have assessed the presence of fosfomycin in river water or sewage. However, given the findings from a previous study in Curitiba city, which detected fluoroquinolone residues, it is reasonable to consider that a similar occurrence may be possible with fosfomycin^30^. Nonetheless, the genetic transfer of resistance mechanisms is a more plausible explanation for the development of resistance due to the presence of drugs in water. In our analysis, when we examined the correlation between the percentage of environmental isolates resistant to fosfomycin and the different sanitary districts, we found no significant association between environmental resistance and resistance observed in clinical isolates.

In a multicentric study conducted in Italy, the mean resistance rate against fosfomycin was 9.7% (range 7.1–11.3), higher among ESBL-producing bacteria when compared with non-ESBL-producing strains (10.8% vs. 7.9%, respectively; P < 0.001)^31^. In a study conducted in Portugal, out of the 19,186 *E. coli* isolates, 100 were fosfomycin resistant (0.5%), out of which 15 carried a *fos*A-like gene (15%)^32^. In Egypt, a study found a high percentage of fosfomycin resistance (37/96; 38.5%), which was reported among uropathogenic *E. coli* isolates^33^. In Belgium, fosfomycin and ciprofloxacin displayed higher resistance rates in subjects older than 80 years (18%–24% in females; 25%–35% in males, respectively)^34^. In Brazil, *E. coli* showed the highest rate of susceptibility to fosfomycin (98.1%)^35^.

Furthermore, we emphasize that a prior study conducted in Curitiba revealed that even though sewage effluents undergo treatment, the efficacy of this treatment in eliminating antibiotic-resistant microorganisms and antibiotic residues is limited. Additionally, another study in Curitiba demonstrated elevated antibiotic concentrations in certain rivers, indicating a substantial level of contamination from domestic sewage. This underscores the importance of proper sanitation practices and highlights how contaminated rivers can evolve into a natural reservoir of antibiotic resistance^30,36^.

Although a greater number of ciprofloxacin-resistant strains were isolated from both clinical and environmental strains (5.6% and 10.7%, respectively), fosfomycin resistance was more associated with the presence of ESBL in both groups (17.3% and 22.7%, respectively). Fosfomycin resistance rates among ESBL-producing Enterobacterales have been reported in previous studies and are increasing globally^37^. The three major mechanisms of fosfomycin resistance include: 1) reduced antibiotic uptake; 2) modification of the antibiotic target and; 3) inactivation by enzymes that can modify fosfomycin^38^. The *fos*A gene, a fosfomycin-modifying gene, was first reported in a plasmid of clinical isolates of *Serratia marcescens* in 1980^39^. Plasmids carrying fosfomycin-modifying genes commonly harbor additional resistance genes, mainly the ESBL CTX-M gene, and selective pressure by other antimicrobial agents increases the risk of fosfomycin resistance^40^.

In this study, we focused solely on non-susceptible isolates of *E. coli*, specifically those that were ESBL-positive and non-susceptible to ciprofloxacin. This selection bias was intentional and does not necessarily reflect the susceptibility profile of fosfomycin in the broader population of *E. coli* isolates associated with urinary tract infections (UTIs). We highlight that our study did not primarily address molecular analysis of ESBL and ciprofloxacin resistance, although such analysis would be a valuable complement to the research. Moreover, the CTX-M genotype has been the most prevalent ESBL genotype in Curitiba over the past decade, providing insight into the local patterns of ESBL resistance^30^.

## CONCLUSIONS

In this study, we identified *E. coli* strains with fosfomycin resistance in approximately 5% to 6.5% of clinical urine samples (as per CLSI and EUCAST standards, respectively), as well as in 11.1% to 13.8% of environmental river water samples (according to CLSI and EUCAST, respectively). These resistant strains were found distributed among nine out of the 10 sanitary districts and four out of the six main rivers in Curitiba City. We highlight that further research is needed to explore additional reservoirs and to continuously monitor the occurrence and resistance profiles. Moreover, resistance was higher in environmental isolates than in clinical. This discrepancy is an important issue for future discussions and interventions and contributes to our understanding of the dynamics of antimicrobial resistance.
